# Supplementary figures and images for: Genome-wide phage susceptibility analysis in Acinetobacter baumannii reveals capsule modulation strategies that determine phage infectivity
Source: PLoS Pathog. 2023 Jun 8;19(6):e1010928. doi: 10.1371/journal.ppat.1010928 (PMC10249906; doi:10.1371/journal.ppat.1010928)

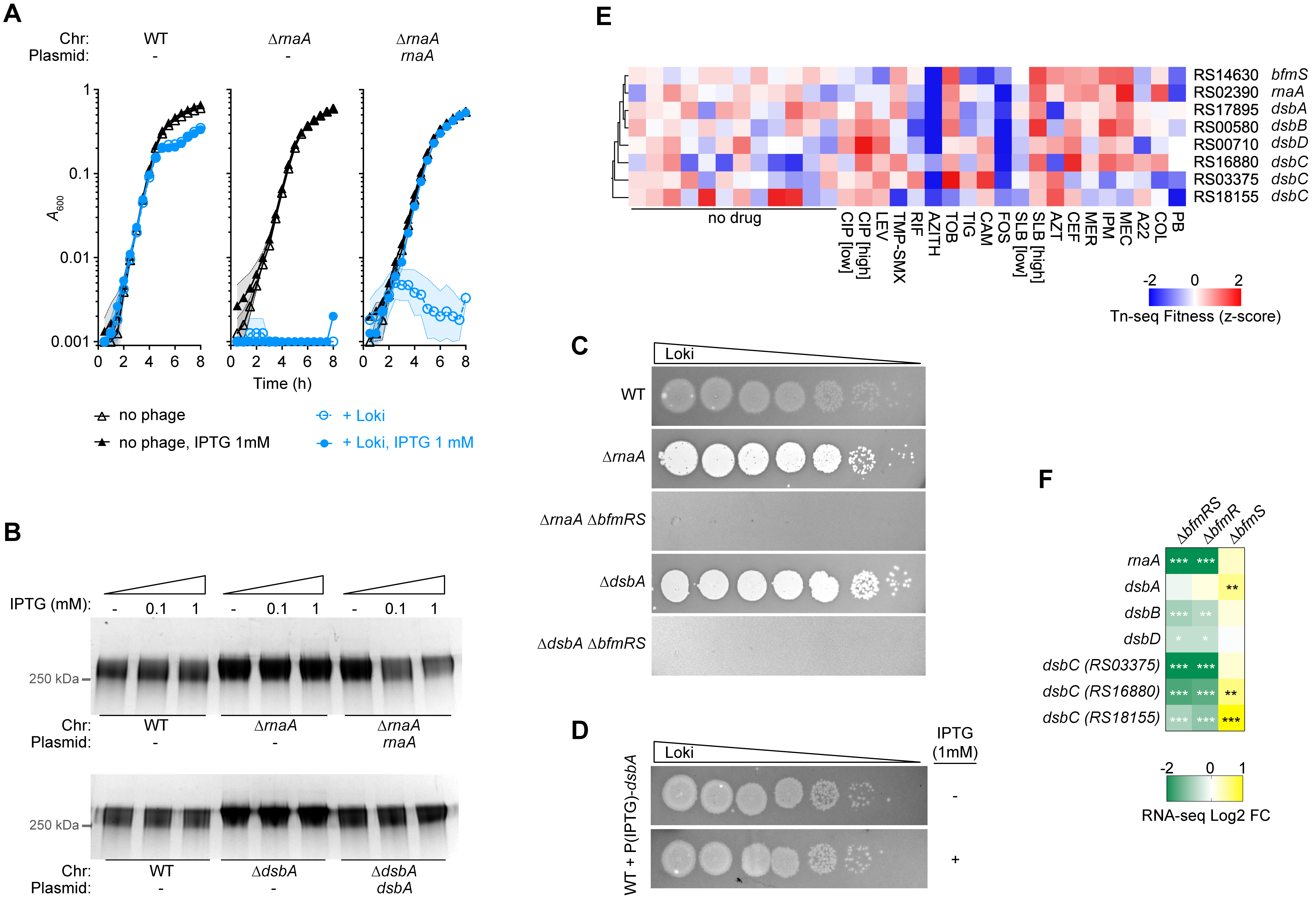

Supplement: S1 Fig — (A) Liquid Loki challenge assays show that overexpression of reintroduced rnaA by a high inducer concentration (1mM IPTG) not only reverses the ∆rnaA phage susceptibility defect, but causes complete non-susceptibility to the phage. “Chr” indicates the chromosomal mutation; “Plasmid” denotes the gene reintroduced under P(IPTG) control via vector pYDE152;—indicates vector with no reintroduced gene. Bacteria were cultured with or without Loki (MOI 1) and IPTG (1mM) as indicated. Data are presented as in Fig 1A (n = 3). (B) Complementation analysis showing reversibility of capsule deficiency in ∆rnaA and ∆dsbA strains. Capsular polysaccharide levels were analyzed by SDS-PAGE/Alcian Blue after growth with the noted amount of IPTG to induce the reintroduced gene. (C-D) Plaque formation assays. Loki was spotted onto bottom agar containing WT bacteria with the indicated genotype. In D, bacteria harbored pJE101 (IPTG-dependent dsbA), and plates contained 0 or 1mM IPTG. (E) Cluster analysis of Tn-seq phenotypic signatures of bfmS, rnaA, and dsb genes. Heat map shows normalized Tn-seq fitness in z-score units for transposon mutants in each gene (rows) grown in different conditions (columns) [41]. Dendrogram at left shows relationship between signatures based on hierarchical clustering. (F) Heat map shows change in RNA-seq transcript levels in the indicated BfmRS mutant compared to WT control. Data were extracted from previous datasets [35] and log2 fold change values displayed as heat map using Prism 9. P values: *, ≤0.05; **, ≤0.01; ***, <0.001. (TIF) [file ppat.1010928.s001.tif]

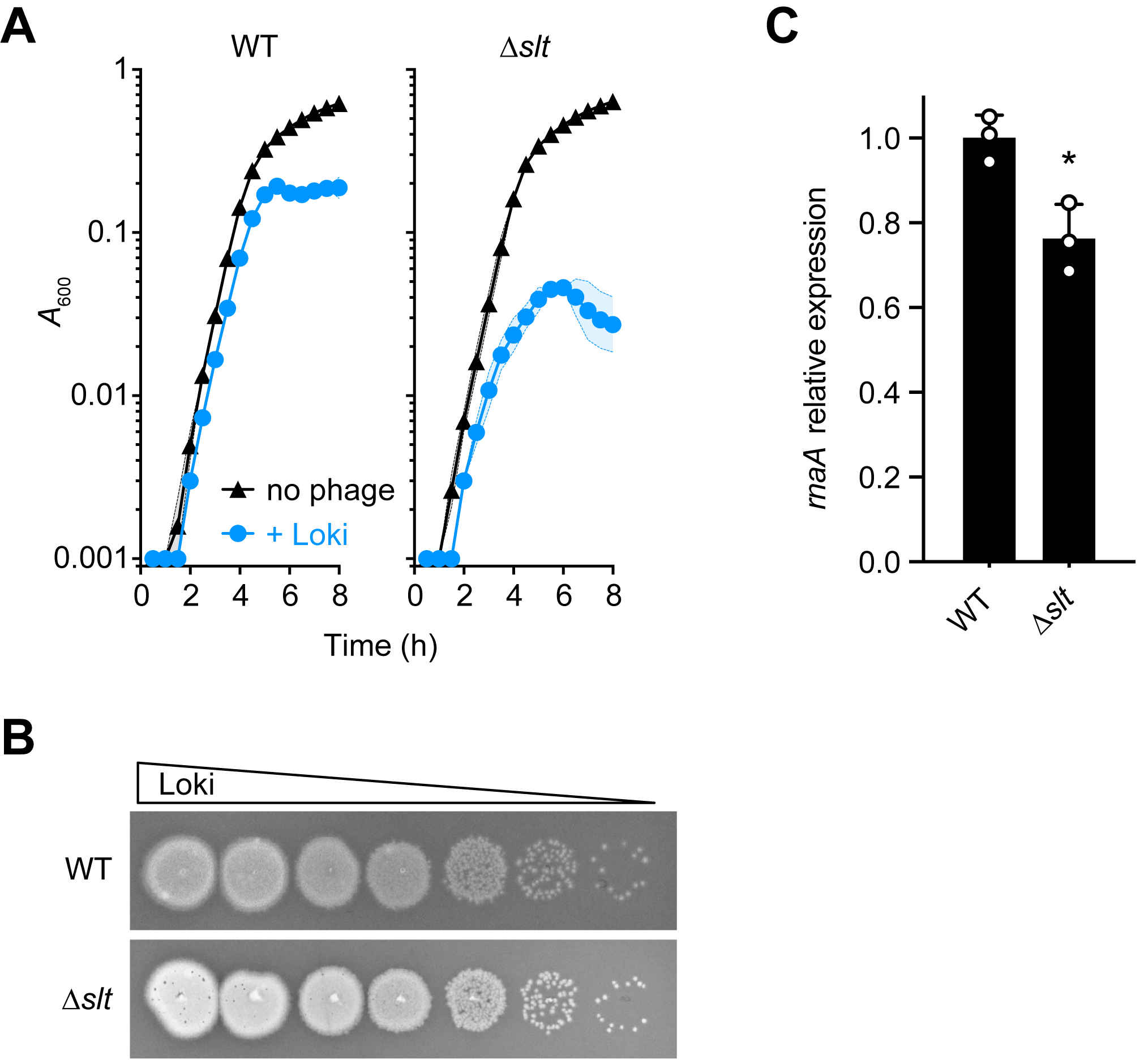

Supplement: S2 Fig — (A) Liquid challenge assay with WT or ∆slt bacteria cultured with or without Loki (MOI 1). Data are presented as in Fig 1A (n = 3). (B) Plaque formation assay with Loki spotted onto top agar with WT and ∆slt bacteria. (C) Measurement of rnaA transcript levels in WT vs ∆slt via qRT-PCR. Bars show mean fold change vs WT ± s.d. (n = 3); analyzed by unpaired t-test. *, P ≤ 0.05. (TIF) [file ppat.1010928.s002.tif]

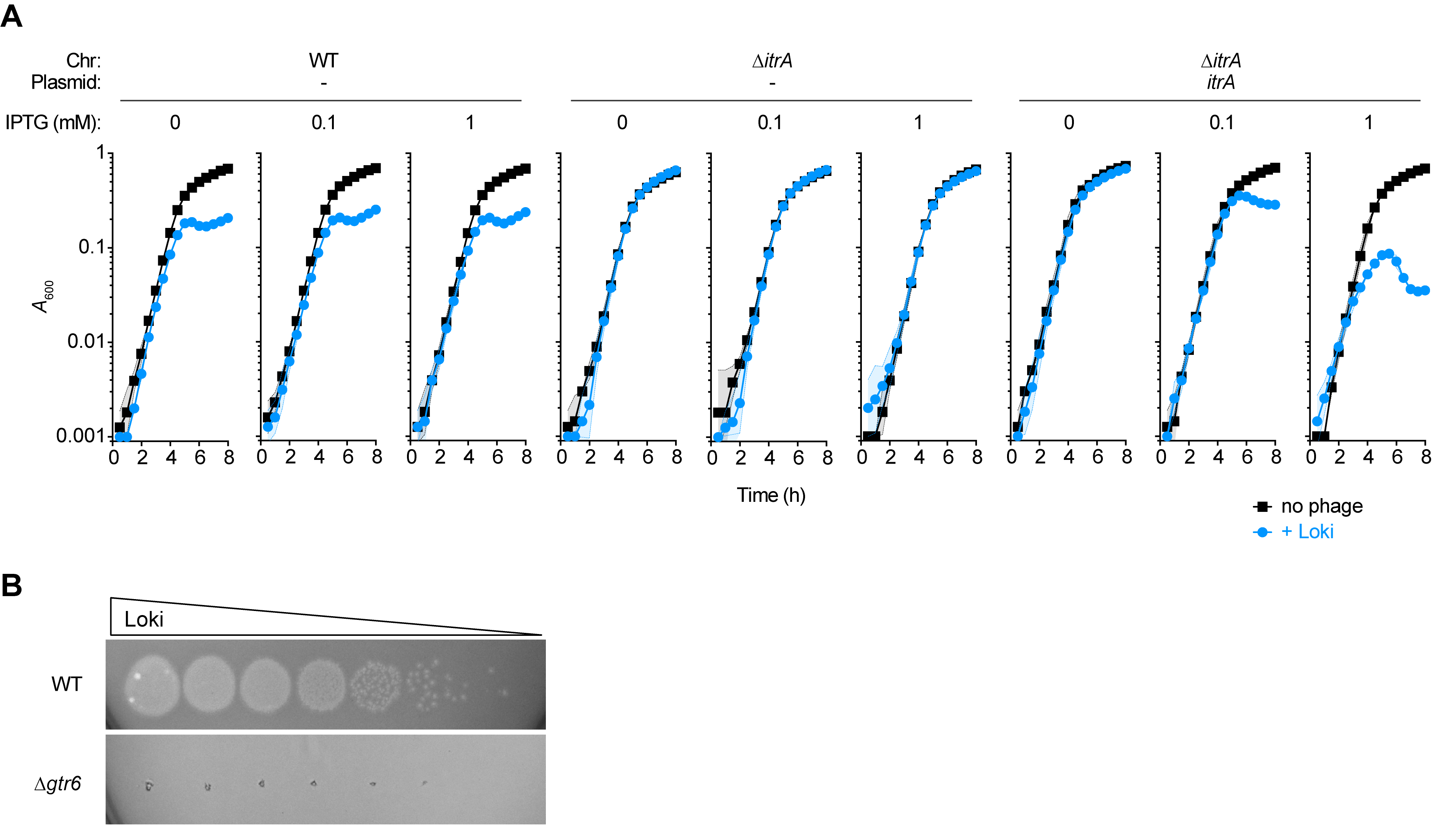

Supplement: S3 Fig — (A) Bacteria of the noted genotype were cultured with the indicated level of IPTG with or without Loki at initial MOI of 1. Growth was measured as in Fig 1A (n = 3). (B) Plaque formation assay with Loki spotted on 17978 WT and isogenic ∆gtr6 mutant. (TIF) [file ppat.1010928.s003.tif]

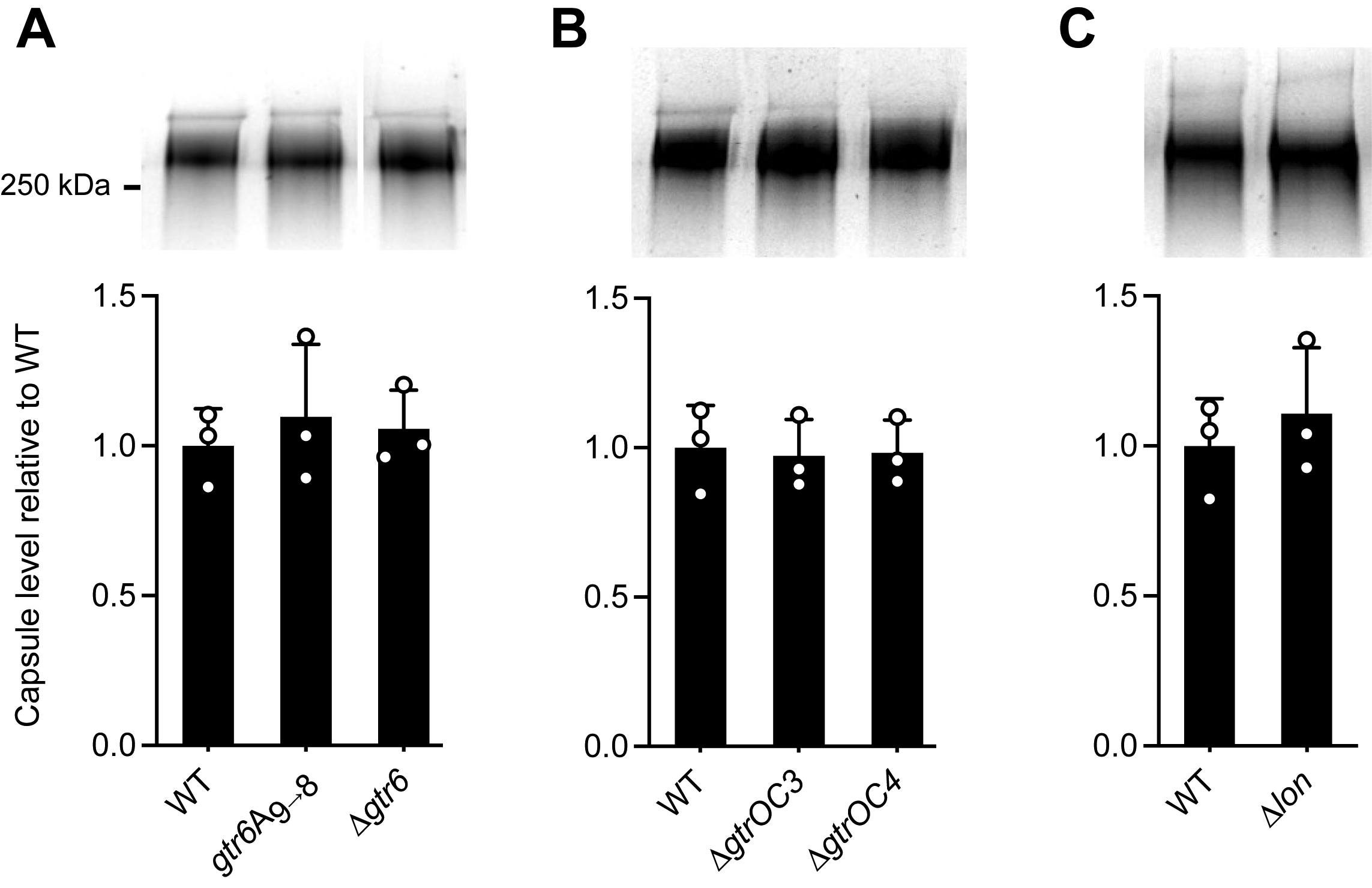

Supplement: S4 Fig — Bars show mean capsule levels ± s.d. (n = 3). Lanes from representative gels are shown above each graph. No significant difference among means by one-way ANOVA (A, P = 0.797; B, P = 0.963) or by t test (C, P = 0.531). (TIF) [file ppat.1010928.s004.tif]

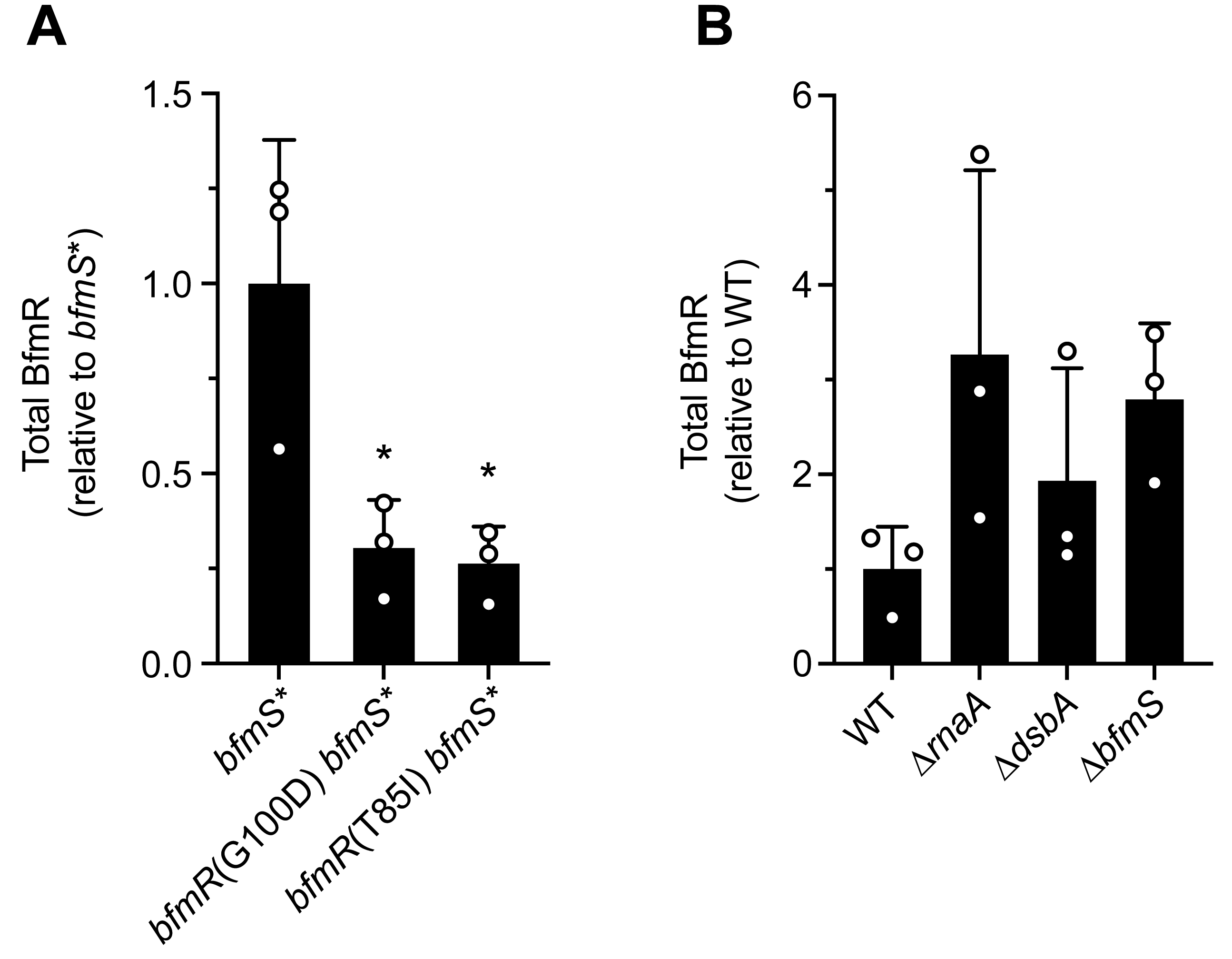

Supplement: S5 Fig — Total BfmR levels were quantified from anti-BfmR Phos-tag western blots by combining the signal from phosphorylated and unphosphorylated forms of BfmR, and normalizing to total protein in the sample as determined by SYPRO Ruby staining. (A) Graph corresponds to the blot shown in Fig 5E. (B) Graph corresponds to the blot shown in Fig 7D. Bars show mean ± s.d. (n = 3), analyzed by one-way ANOVA with Dunnett’s multiple comparisons test (mutant vs WT). *, P value ≤ 0.05. (TIF) [file ppat.1010928.s005.tif]

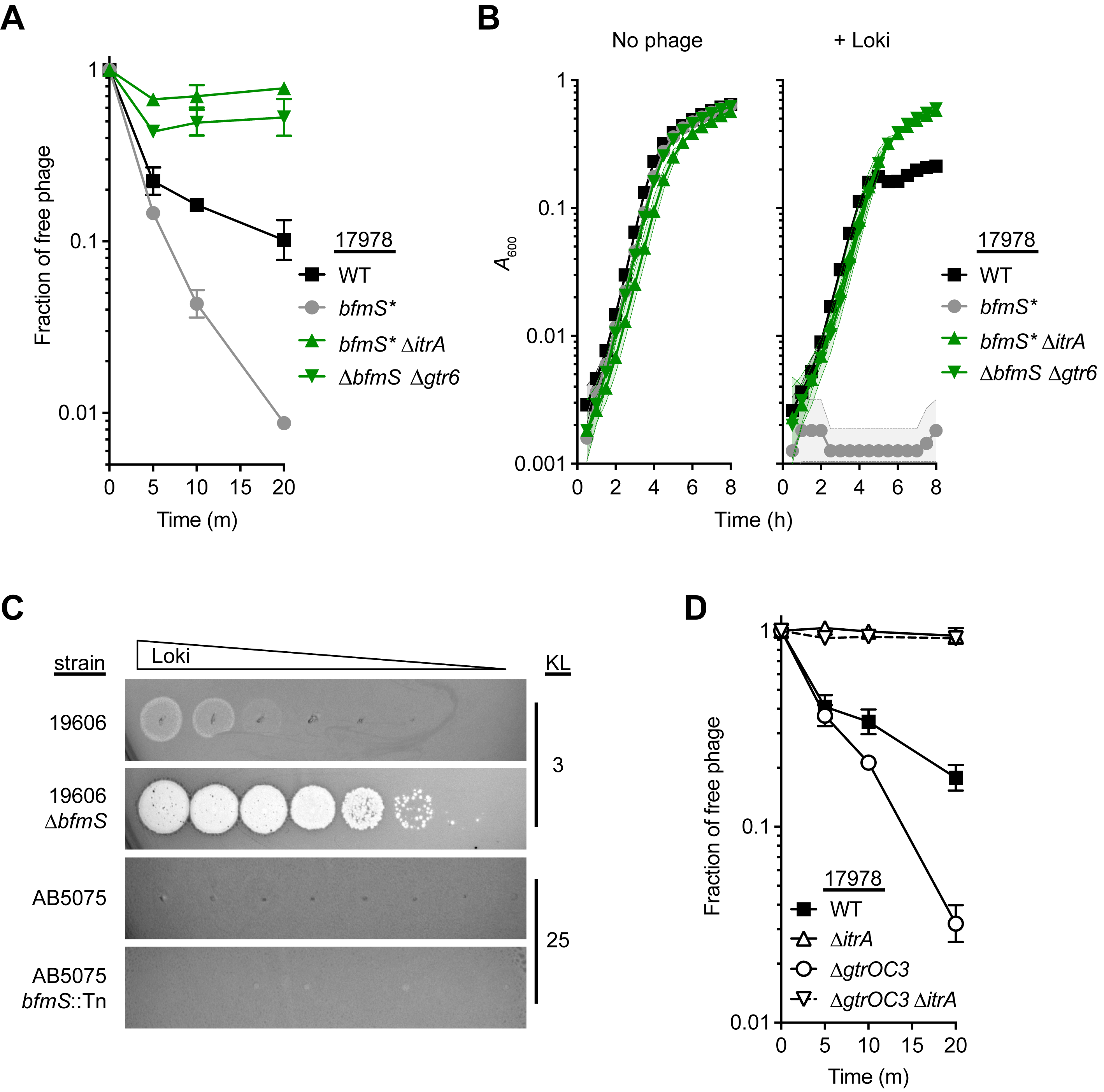

Supplement: S6 Fig — (A, D) Phage adsorption assays showing that enhancement of Loki adsorption by bfmS (A) or gtrOC3 (D) mutations depends on capsule. Data presented as in Fig 4 (n = 3). (B) Liquid challenge assays. The indicated bacteria were cultured with or without Loki (MOI 1). Data are presented as in Fig 1A (n = 3). (C) Plaque formation assays with Loki spotted on A. baumannii strains, and their corresponding bfmS-null derivative, harboring the indicated K locus. (TIF) [file ppat.1010928.s006.tif]

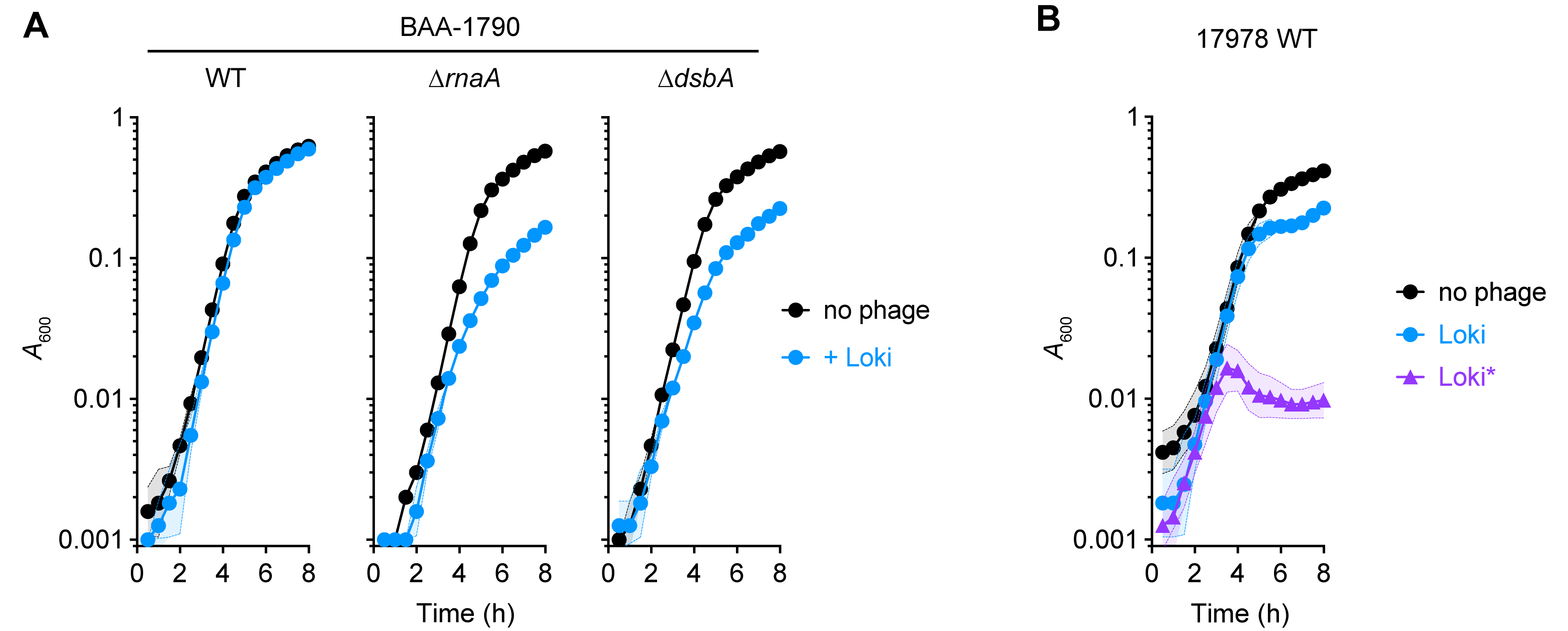

Supplement: S7 Fig — (A) BAA-1790 WT and the indicated isogenic mutants were challenged with Loki. (B) 17978 WT was challenged with Loki, the virulent Loki* derivative, or no phage control. Data points show geometric mean ± s.d. (n = 3). (TIF) [file ppat.1010928.s007.tif]

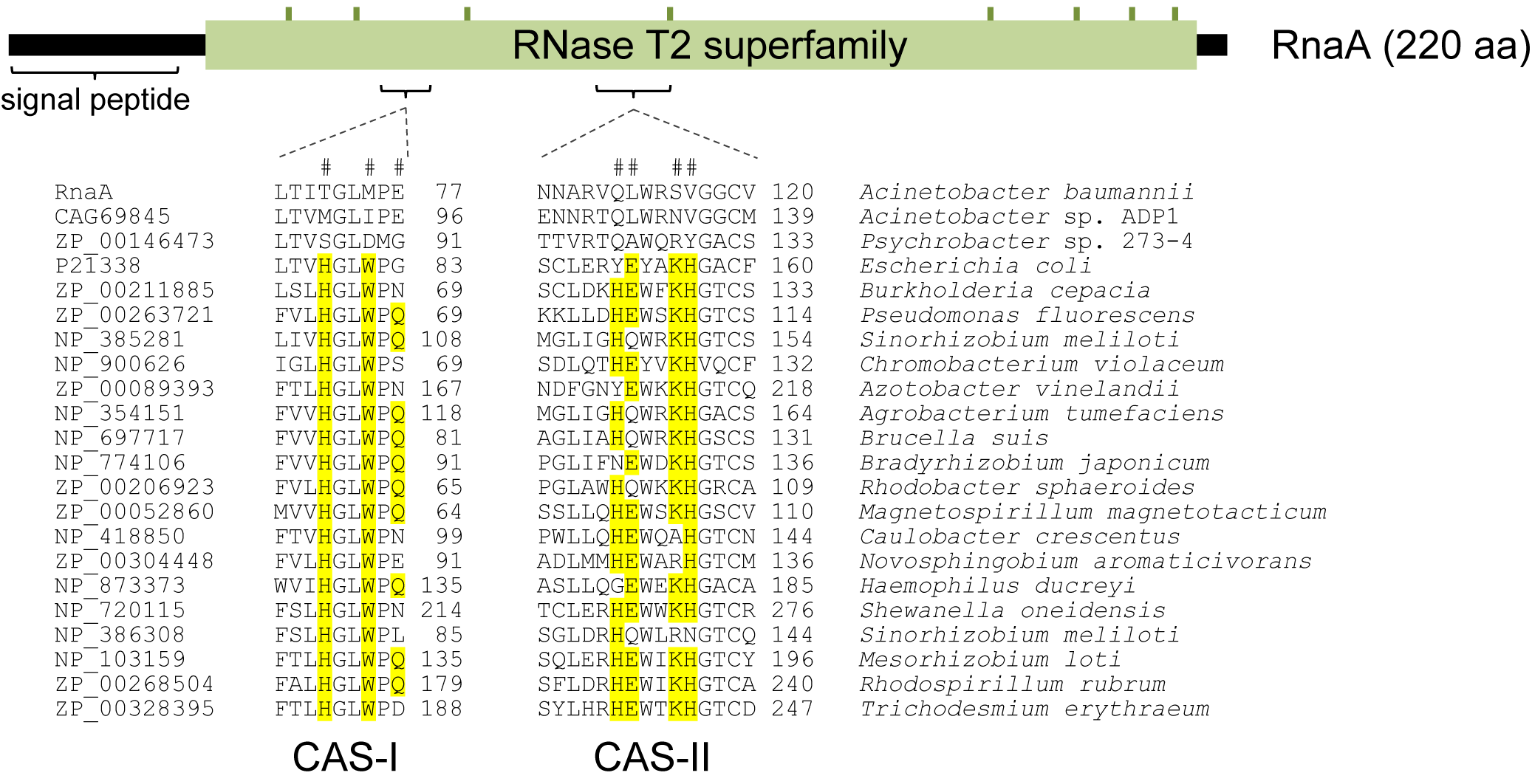

Supplement: S8 Fig — Diagram shows analysis of conserved domains on RnaA. RNase_T2 domain, CAS motifs, and active site positions were identified using the NCBI Conserved Domain Database [87]. Signal peptide was identified using SignalP [88]. Vertical green lines represent conserved cysteine residues within the RnaA RNase_T2 domain. Alignment shows RNase T2 family homologs across diverse bacterial species. Within the conserved active site motifs (CAS-I and CAS-II), the active sites are indicated by #, and the most commonly found residues at these sites are highlighted. (TIF) [file ppat.1010928.s008.tif]

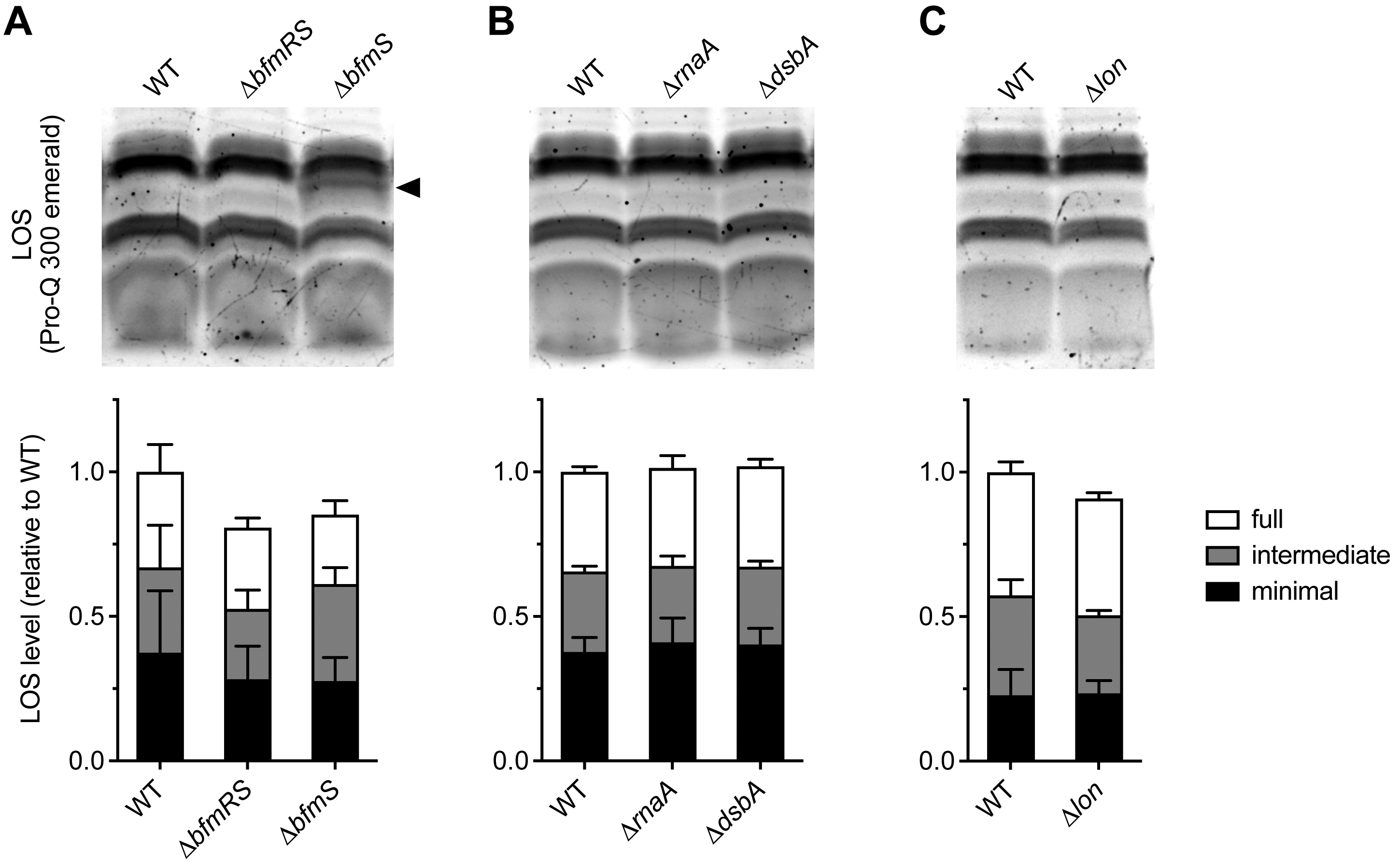

Supplement: S9 Fig — LOS production in cell lysates of mutants in bfmRS (A), rnaA and dsbA (B), and lon (C) was analyzed in comparison with WT control via Pro-Q 300 emerald staining. Representative gels are shown in top panels. Arrowhead indicates location of novel intermediate band associated with ∆bfmS (A). Quantification is shown in bottom panels. Bars show mean ± s.d. (n = 3). Within each set of strains, no significant difference (P > 0.05) observed in total LOS levels by one-way ANOVA (A, B) or unpaired t-test (C). (TIF) [file ppat.1010928.s009.tif]

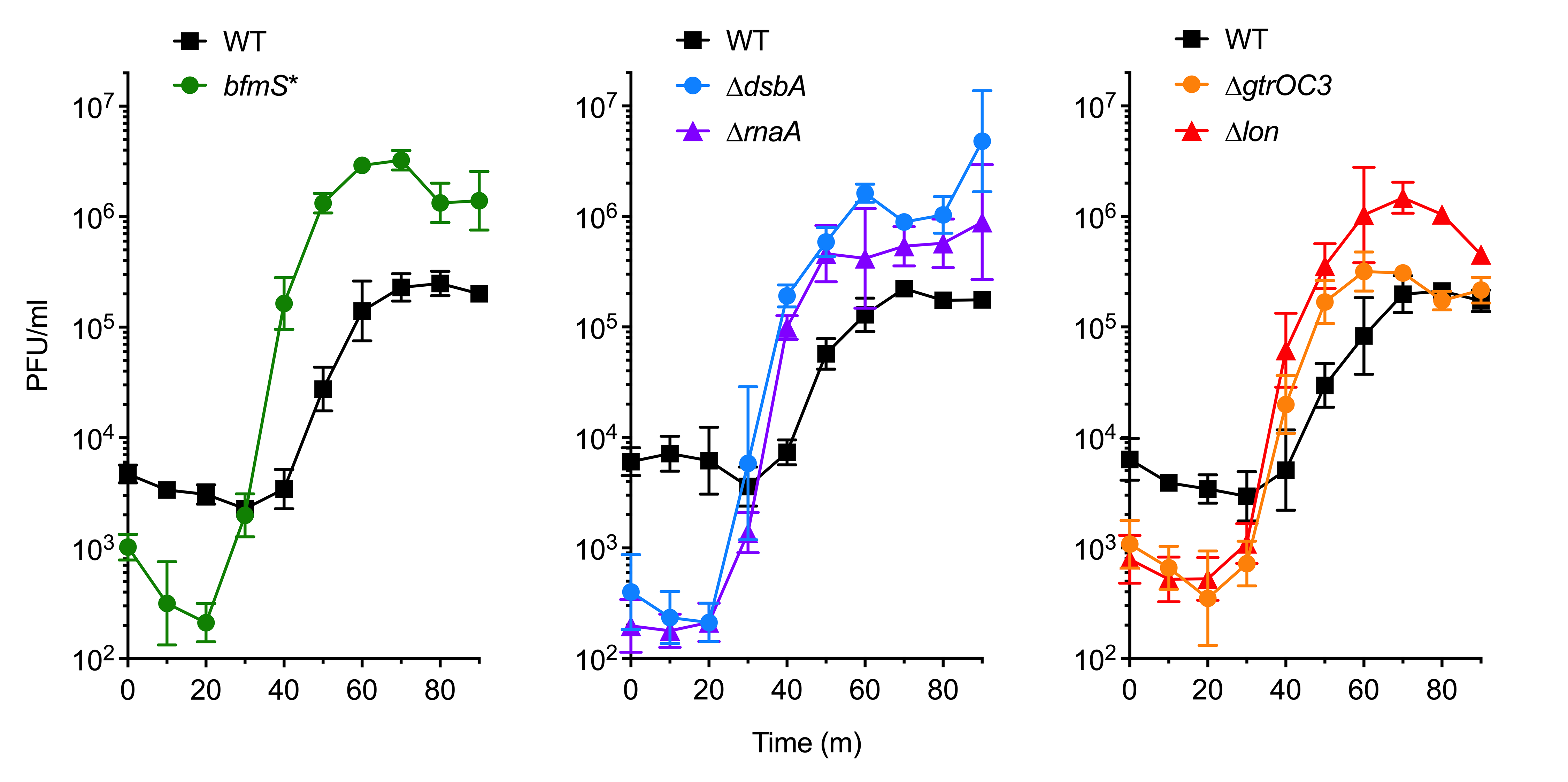

Supplement: S10 Fig — Data points show geometric mean PFU/ml ± s.d. (n = 4). Infected cell number (used in burst size calculations) was approximately 1–1.5 x 10^4^ per ml. (TIFF) [file ppat.1010928.s010.tiff]
