# Supplementary material for: Genome-wide phage susceptibility analysis in Acinetobacter baumannii reveals capsule modulation strategies that determine phage infectivity
Source: PLoS Pathog. 2023 Jun 8;19(6):e1010928. doi: 10.1371/journal.ppat.1010928 (PMC10249906; doi:10.1371/journal.ppat.1010928)
Supplement: S1 Table — (PDF) [file ppat.1010928.s011.pdf]

S1 Table. Strains, plasmids, and primers used in this study

Strains

Designation	Genotype or description	Strain ID	Reference
A. baumannii			
17978	cerebrospinal fluid isolate, AbaAL44$^+$ ("UN") type, ATCC	EGA83	[1, 2]
17978 *bfmS**	ATCC 17978 *bfmS*(G467Dfs*19)	EGA127	[3]
17978 Δ*bfmS*	ATCC 17978 Δ*bfmS*::*aacC1*	EGA195	[3]
17978 Δ*bfmRS*	ATCC 17978 Δ*bfmRS*::*aacC1*	EGA495	[7]
17978 Δ*itrA*	ATCC 17978 Δ*itrA*	EGA295	[3]
17978 Δ*wzc*	ATCC 17978 Δ*wzc::aacC1*, isolate 27	EGA106-27	[3]
17978 Δ*wzc wzc*$^+$	ATCC 17978 Δ*wzc::aacC1*, *wzc*$^+$ by marker rescue of EGA106-27	EGA260	[3]
17978 Δ*dsbA*	ATCC 17978 Δ*dsbA*	NRA134	This work
17978 Δ*lon*	ATCC 17978 Δ*lon*	JBA154	This work
17978 Δ*gtrOC3*	ATCC 17978 Δ*gtrOC3*	JBA160	This work
17978 Δ*gtrOC4*	ATCC 17978 Δ*gtrOC4*	JBA161	This work
17978 Δ*gtr6*	ATCC 17978 Δ*gtr6*	JBA202	This work
17978 Δ*rnaA*	ATCC 17978 Δ*rnaA*	JBA211	This work
17978 Δ*slt*	ATCC 17978 Δ*slt*	EGA520	This work
17978 Δ*rnaA*Δ*bfmRS*	ATCC 17978 Δ*rnaA* Δ*bfmRS*	JBA234	This work
17978 Δ*dsbA*Δ*bfmRS*	ATCC 17978 Δ*dsbA* Δ*bfmRS*	JBA235	This work
17978 *bfmS**Δ*itrA*	ATCC 17978 *bfmS*(G467Dfs*19) Δ*itrA*	JBA210	This work
17978 Δ*bfmS**Δ*gtr6*	ATCC 17978 Δ*bfmS*::*aacC1* Δ*gtr6*	JBA208	This work
17978 Δ*gtrOC3*Δ*itrA*	ATCC 17978 Δ*gtrOC3* Δ*itrA*	JBA212	This work
17978 Δ*gtrOC3*Δ*gtr6*	ATCC 17978 Δ*gtrOC3* Δ*gtr6*	JBA215	This work
17978 Δ*dsbA/-*	NRA134 with pYDE152	JBA172	This work
17978 Δ*dsbA/dsbA*	NRA134 with pJE101 (P(IPTG)-*dsbA*)	JBA168	This work
17978 Δ*lon/-*	JBA154 with pYDE152	JBA174	This work
17978 Δ*lon/lon*	JBA154 with pJE103 (P(IPTG)-*lon*)	JBA170	This work
17978 Δ*gtrOC3/-*	JBA160 with pYDE152	JBA171	This work
17978 Δ*gtrOC3/ gtrOC3*	JBA160 with pJE100 (P(IPTG)-*gtrOC3*)	JBA167	This work
17978 Δ*gtrOC4/-*	JBA161 with pYDE152	JBA173	This work
17978 Δ*gtrOC4/ gtrOC4*	JBA161 with pJE102 (P(IPTG)-*gtrOC4*)	JBA169	This work
17978 Δ*rnaA/-*	JBA211 with pYDE152	JBA218	This work
17978 Δ*rnaA/-*	JBA211 with pJE127 (P(IPTG)-*rnaA*)	JBA217	This work
17978 Δ*itrA/-*	EGA295 with pYDE152	JBA270	This work
17978 Δ*itrA/itrA*	EGA295 with pJE172 (P(IPTG)-*itrA*)	JBA271	This work
17978 + P(IPTG)-*rnaA*	ATCC 17978 WT with pJE127 (P(IPTG)-*rnaA*)	JBA266	This work
17978 *bfmS** + P(IPTG)-*rnaA*	EGA127 with pJE127 (P(IPTG)-*rnaA*)	JBA256	This work
17978 + P(IPTG)-*dsbA*	ATCC 17978 WT with pJE101 (P(IPTG)-*dsbA*)	JBA349	This work
17978 *gtr6*440$A_{9\rightarrow8}$	Loki non-susceptible derivative of WT ATCC 17978 (*gtr6*440$A_{9\rightarrow8}$), 2 independent isolates	JBA149, JBA267	This work
17978 *wzx*377$T_{9\rightarrow10}$	Loki non-susceptible derivative of WT ATCC 17978 (*wzx*377$T_{9\rightarrow10}$)	JBA150	This work
17978 *bfmS** *gtr6*440$A_{9\rightarrow8}$	Loki non-susceptible derivative of EGA127 (*gtr6*440$A_{9\rightarrow8}$)	JBA151	This work
17978 *bfmS** *bfmR*(G100D)	Loki non-susceptible derivative of EGA127 [*bfmR*(G100D)]	JBA152	This work

S1 Table (continued)

17978 *bfmS** *bfmR*(T85I)	Loki non-susceptible derivative of EGA127 [*bfmR*(T85I)]	JBA153	This work
17978 *bfmS** *gtr6*384$A_{8\rightarrow7}$	Loki non-susceptible derivative of EGA127 (*gtr6*384$A_{8\rightarrow7}$)	JBA261	This work
17978 Δ*bfmS* *gtr6*440$A_{9\rightarrow8}$	Loki non-susceptible derivative of EGA195 (*gtr6*440$A_{9\rightarrow8}$)	JBA268	This work
17978 *gtr6*440$A_{9\rightarrow8}$/-	JBA149 with pYDE152	JBA204	This work
17978 *gtr6*440$A_{9\rightarrow8}$/*gtr6*	JBA149 with pJE121 (P(IPTG)-*gtr6*)	JBA205	This work
17978 *10840*p-GFP	ATCC 17978 WT with pEGE246 (*10840*p-GFP)	EGA616	[7]
17978 Δ*bfmRS* *10840*p-GFP	EGA495 with pEGE246 (*18040*p-GFP)	NRA104	This work
17978 Δ*dsbA* *10840*p-GFP	NRA134 with pEGE246 (*18040*p-GFP)	NRA178	This work
17978 Δ*dsbA*Δ*bfmRS* *10840*p-GFP	JBA235 with pEGE246 (*18040*p-GFP)	NRA236	This work
17978 Δ*rnaA* *10840*p-GFP	JBA211 with pEGE246 (*18040*p-GFP)	NRA238	This work
17978 Δ*rnaA*Δ*bfmRS* *10840*p-GFP	JBA234 with pEGE246 (*18040*p-GFP)	NRA240	This work
17978 *adc*p-GFP	ATCC 17978 WT with pEGE313 (*adc*p-GFP)	EGA786	[7]
17978 Δ*dsbA* *adc*p-GFP	NRA134 with pEGE313 (*adc*p-GFP)	NRA237	This work
17978 Δ*rnaA* *adc*p-GFP	JBA211 with pEGE313 (*adc*p-GFP)	NRA239	This work
17961	blood isolate, ATCC	EGA56	[1]
19606	urine isolate, ATCC	EGA2M	[1]
19606 Δ*bfmS*	ATCC 19606 Δ*bfmS*::*aacC1*	EGA216	[3]
AB5075	bone isolate/osteomyelitis, AB5075-UW	EGA714	[4]
AB5075 *bfmS*::Tn	AB5075-UW *bfmS*115::T26	EGA725	[4]
BAA-1790	sputum isolate (2008, Washington, DC), ATCC	JBA394	[6]
BAA-1790 Δ*rnaA*	BAA-1790 Δ*rnaA*, via allele exchange with pJE193	JBA395	This work
BAA-1790 Δ*dsbA*	BAA-1790 Δ*dsbA*, via allele exchange with pJE195	JBA396	This work
EGA10	wound isolate, Tufts University Medical Center, ST2	EGA10	This work
EGA65	blood isolate, Tufts University Medical Center, ST2	EGA65	This work
EGA10/-	EGA10 with pYDE152	JBA246	This work
EGA10/*gtr6*	EGA10 with pJE121 (P(IPTG)-*gtr6*)	JBA247	This work
EGA65/-	EGA65 with pYDE152	JBA248	This work
EGA65/*gtr6*	EGA65 with pJE121 (P(IPTG)-*gtr6*)	JBA249	This work
E. coli			
DH5α	*supE44* Δ*lacU169* (φ80lacZΔM15) *hsdR17 recA1 endA1 gyrA96 thi*-1 *relA1*	EGE1	[8]
DH5λpir	DH5α (λpir) *tet*::Mu *recA*	EGE4	[9]
XL1-blue	*recA1 endA1 gyrA96 thi-1 hsdR17 supE44 relA1 lac* [F' *proAB lacI*^{q}ZΔM15 Tn10 Tc^r)]	AFE81	Stratagene
Bacteriophage			
Loki	vB_AbaS_Loki, lytic *Acinetobacter* siphovirus isolated from activated sewage sludge	Loki	[10]
Loki*	Derivative of Loki showing enhanced virulence vs 17978 and BAA-1790	JBΦ-15C	This work

S1 Table (continued)

Plasmids

Plasmid	Description	Reference
pUC18	*oriColE1* MCS Cb^r	[11]
pSR47S	Conditionally replicating allele exchange plasmid (*oriTRP4 oriR6K sacB* Km^r)	[12]
pJB4648	Conditionally replicating allele exchange plasmid (*oriTRP4 oriR6K sacB* Gm^r)	[12]
pEGE305	P(IPTG) shuttle vector (*ori-pBR322 ori*-pWH1277 *bla*::*lacI*^{q}-T5*lac*P Tc^r)	[7]
pYDE152	P(IPTG) shuttle vector (*ori-pBR322 ori-pWH1277 bla*::*lacI*^{q}-T5*lac*P-MCS Tc^r)	[13]
pDL1100	*Himar1 mariner* (Km^r) delivery plasmid, C9 transposase (*ori-pSC101* Cb^r)	[14]
pJE89	pUC18 containing homology arm upstream of *gtrOC3*	This work
pJE90	pUC18 containing homology arm downstream of *gtrOC3*	This work
pJE93	pJB4648::Δ*gtrOC3* allele exchange construct	This work
pJE91	pUC18 containing homology arm upstream of *gtrOC4*	This work
pJE92	pUC18 containing homology arm downstream of *gtrOC4*	This work
pJE94	pJB4648::Δ*gtrOC4* allele exchange construct	This work
pJE114	pUC18 containing homology arm upstream of *gtr6*	This work
pJE115	pUC18 containing homology arm downstream of *gtr6*	This work
pJE118	pJB4648::Δ*gtr6* allele exchange construct	This work
pJE122	pUC18 containing homology arm upstream of *rnaA*	This work
pJE123	pUC18 containing homology arm downstream of *rnaA*	This work
pJE124	pJB4648::Δ*rnaA* allele exchange construct	This work
pEGE242	pSR47S::Δ*slt* allele exchange construct	This work
pEGE133	pSR47S::Δ*bfmRS*::*aacC1* allele exchange construct	[3]
pEGE125	pSR47S::Δ*bfmS*::*aacC1* allele exchange construct	[3]
pEGE181	pSR47S::Δ*itrA* allele exchange construct	[3]
pJE95	pUC18::*gtrOC3*	This work
pJE99	pUC18::*gtrOC4*	This work
pJE120	pUC18::*gtr6*	This work
pJE171	pUC18::*itrA*	This work
pJE126	pUC18::*rnaA*	This work
pJE97	pUC18::*lon*	This work
pJE98	pUC18::*dsbA*	This work
pJE100	pYDE152::*gtrOC3* (P(IPTG)-*gtrOC3*)	This work
pJE102	pYDE152::*gtrOC4* (P(IPTG)-*gtrOC4*)	This work
pJE121	pYDE152::*gtr6* (P(IPTG)-*gtr6*)	This work
pJE172	pYDE152::*itrA* (P(IPTG)-*itrA*)	This work
pJE127	pYDE152::*rnaA* (P(IPTG)-*rnaA*)	This work
pJE103	pYDE152::*lon* (P(IPTG)-*lon*)	This work
pJE101	pYDE152::*dsbA* (P(IPTG)-*dsbA*)	This work
pEGE246	*ACX60_RS18040*p-*gfpmut3* reporter plasmid (*ori-pBR322 ori*-pWH1277, Tc^r)	[7]
pEGE313	*adc*p-*gfpmut3* reporter plasmid (*ori-pBR322 ori*-pWH1277, Tc^r)	[7]
pJE193	pSR47S::Δ*rnaA*(BAA-1790) allele exchange construct	This work
pJE195	pSR47S::Δ*dsbA*(BAA-1790) allele exchange construct	This work

S1 Table (continued)

Oligonucleotide primers

Primer name	Sequence (5’ – 3’; restriction site underlined if present)	RE site(s)
Complementation and localization experiments		
dsbA-F	CGAGCTCTAGAGGAAAAGCTGTAACAATGAAA	SacI
dsbA-R	AAAACTGCAGGCAATAAACTATTATTTTGCCTTAC	PstI
lon-F	CGAGCTCATTAGGAGTGCCCATGTCTG	SacI
lon-R	AAAACTGCAGGTGAATTAGTGACGCGCTGCTTTTG	PstI
gtrOC3-F	CGAGCTCGCTAAAGGACGTTATAAGTTATGAAT	SacI
gtrOC3-R	AAAACTGCAGGCTTGCCTACTTATTTTTATCTTTATTC	PstI
gtrOC4-F	CGAGCTCGATAAAAATAAGTAGGCAAGCTATGAAAATTG	SacI
gtrOC4-R	AAAACTGCAGTAATTAAGTTTTCAGCAGGCCTTTAT	PstI
gtr6-F	CGAGCTCTAAGGTTACTATATGAAAATTGGATTG	SacI
gtr6-R	AAAACTGCAGCACCTACGACATCATTATTAGGTACA	PstI
itrA-F	CGAGCTCGACTGTTACCAGCGAATTTATTA	SacI
itrA-R	TGTACTGCAGCCGGCTACTGGTAAAACTG	PstI
rnaA-F	CGAGCTCAATGATGGAACATTCTTTTAATTG	SacI
rnaA-R	AAAACTGCAGGGTTATTGCTCTTAATAACTGCCT	PstI
Gene deletion		
gtrOC4-upF	ACGCGTCGACGTGCCCGAGTTTTGCTTATC	SalI
gtrOC4-upR	GGGGTACCCTGAACAATTTTCATAGCTTGCCTAC	KpnI
gtrOC4-downF	GGGGTACCGGCCTGCTGAAAACTTAATTATTTTTTGGTG	KpnI
gtrOC4-downR	AAATATGCGGCCGCGTATATGCGCTTTGGCTGGT	NotI
gtrOC3-upF	ACGCGTCGACTGACTTGGCCAAAAAGATCC	SalI
gtrOC3-upR	GGGGTACCACATGGAATTTTATTCATAACTTATAACG	KpnI
gtrOC3-downF	GGGGTACCTAGGCAAGCTATGAAAATTGTTCAGGT	KpnI
gtrOC3-downR	AAATATGCGGCCGCTCAGTTCAGCCTTGGCTTTT	NotI
gtr6-UP-F	ACGCGTCGACGGCATATGCAATCTCAGG	SalI
gtr6-UP-R	GGGGTACCTGTCAATCCAATTTTCATATAGTA	KpnI
gtr6-DOWN-F	GGGGTACCACTATTGGTATTTAATATGTTATCTTGT	KpnI
gtr6-DOWN-R	AAATATGCGGCCGCATGATGCTAGCCCAATCC	NotI
Dlon-up-F	GGATCCTTAGCGCTTCCTTCACG	BamHI
Dlon-up-R	GGTACCCATAATAAGTTCAGACATGGGC	KpnI
Dlon-dwn-F	GGTACCGCAGCGCGTCACTAATTC	KpnI
Dlon-dwn-R	GTCGACCGATGTAAGGCAGGACCC	SalI
rnaA-UP-F	ACGCGTCGACGCGATAGTAATTCTAAAAACACG	SalI
DrnaA-UP-F1790	ACGCGTCGACGCGATAGTAATTCTAAGAATACGG	SalI
rnaA-UP-R	GGGGTACCATATTTCATTTAATTAAAACTTGGTTATG	KpnI
rnaA-DOWN-F	GGGGTACCGGCAGTTATTAAGAGCAATAACCA	KpnI
rnaA-DOWN-R	AAATATGCGGCCGCAGAAGCAGCAGAAGACAAAC	NotI
Dslt-upF	CACCTGGATCCGTCTCACCACGATATCCATTCAC	BamHI
Dslt-upR	CATAGGTACCTTTCATCACACGACTCTTGTTTG	KpnI
Dslt-dwnF	AGTCGGTACCACGCGATCATCACCTTAGTTTTAG	KpnI
Dslt-dwnR	ATCTTGCGGCCGCATTTCACCGCTTGCGATAC	NotI

S1 Table (continued)

qRT-PCR		
wza-F	CTGGCTATTCACTGCACAAAC	
wza-R	CCTGACTCACCCATCACATAAA	
gna-F	GCGGGTAGTAAGTGGAACTTT	
gna-R	AACCTACCTCTTCTGCTTTATGG	
rnaA-qRT-F	GTGCGCTAGACCCTTCTAAAC	
rnaA-qRT-R	ACAGTCAGTTCTGGTGGTTTC	

References

1. Bouvet P, Grimont P. Taxonomy of the Genus *Acinetobacter* with the Recognition of *Acinetobacter baumannii* sp. nov. *Acinetobacter haemolyticus* sp. nov. *Acinetobacter johnsonii* sp. nov. and *Acinetobacter junii* sp. nov. and Emended Descriptions of *Acinetobacter calcoaceticus* and *Acinetobacter lwofii*. International Journal of Systematic Bacteriology. 1986;36(2):228-40.
2. Wijers CDM, Pham L, Menon S, Boyd KL, Noel HR, Skaar EP, et al. Identification of Two Variants of *Acinetobacter baumannii* Strain ATCC 17978 with Distinct Genotypes and Phenotypes. Infect Immun. 2021;89(12):e0045421. Epub 20210830. doi: 10.1128/IAI.00454-21. PubMed PMID: 34460288; PubMed Central PMCID: PMCPMC8594612.
3. Geisinger E, Isberg RR. Antibiotic modulation of capsular exopolysaccharide and virulence in *Acinetobacter baumannii*. PLoS Pathog. 2015;11(2):e1004691. Epub 2015/02/14. doi: 10.1371/journal.ppat.1004691. PubMed PMID: 25679516; PubMed Central PMCID: PMCPMC4334535.
4. Gallagher LA, Ramage E, Weiss EJ, Radey M, Hayden HS, Held KG, et al. Resources for Genetic and Genomic Analysis of Emerging Pathogen *Acinetobacter baumannii*. J Bacteriol. 2015;197(12):2027-35. doi: 10.1128/JB.00131-15. PubMed PMID: 25845845; PubMed Central PMCID: PMCPMC4438207.
5. Gallagher LA, Lee SA, Manoil C. Importance of Core Genome Functions for an Extreme Antibiotic Resistance Trait. MBio. 2017;8(6). Epub 2017/12/14. doi: 10.1128/mBio.01655-17. PubMed PMID: 29233894; PubMed Central PMCID: PMCPMC5727411.
6. Korotetskiy IS, Joubert M, Magabotha SM, Jumagaziyeva AB, Shilov SV, Suldina NA, et al. Complete Genome Sequence of Collection Strain *Acinetobacter baumannii* ATCC BAA-1790, Used as a Model To Study the Antibiotic Resistance Reversion Induced by Iodine-Containing Complexes. Microbiol Resour Announc. 2020;9(3). Epub 20200116. doi: 10.1128/MRA.01467-19. PubMed PMID: 31948971; PubMed Central PMCID: PMCPMC6965589.
7. Geisinger E, Mortman NJ, Vargas-Cuebas G, Tai AK, Isberg RR. A global regulatory system links virulence and antibiotic resistance to envelope homeostasis in *Acinetobacter baumannii*. PLoS Pathog. 2018;14(5):e1007030. Epub 2018/05/26. doi: 10.1371/journal.ppat.1007030. PubMed PMID: 29795704; PubMed Central PMCID: PMCPMC5967708.
8. Hanahan D, Jessee J, Bloom FR. Plasmid transformation of *Escherichia coli* and other bacteria. Methods Enzymol. 1991;204:63-113. Epub 1991/01/01. doi: 0076-6879(91)04006-A [pii]. PubMed PMID: 1943786.
9. Kolter R, Inuzuka M, Helinski DR. Trans-complementation-dependent replication of a low molecular weight origin fragment from plasmid R6K. Cell. 1978;15(4):1199-208. Epub 1978/12/01. doi: 0092-8674(78)90046-6 [pii]. PubMed PMID: 728998.
10. Turner D, Wand ME, Briers Y, Lavigne R, Sutton JM, Reynolds DM. Characterisation and genome sequence of the lytic *Acinetobacter baumannii* bacteriophage vB_AbaS_Loki. PLoS One. 2017;12(2):e0172303. Epub 2017/02/17. doi: 10.1371/journal.pone.0172303. PubMed PMID: 28207864; PubMed Central PMCID: PMCPMC5313236.
11. Yanisch-Perron C, Vieira J, Messing J. Improved M13 phage cloning vectors and host strains: nucleotide sequences of the M13mp18 and pUC19 vectors. Gene. 1985;33(1):103-19. Epub 1985/01/01. PubMed PMID: 2985470.
12. Andrews HL, Vogel JP, Isberg RR. Identification of linked *Legionella pneumophila* genes essential for intracellular growth and evasion of the endocytic pathway. Infect Immun. 1998;66(3):950-8. PubMed PMID: 9488381.
13. Bai J, Dai Y, Farinha A, Tang AY, Syal S, Vargas-Cuebas G, et al. Essential gene analysis in *Acinetobacter baumannii* by high-density transposon mutagenesis and CRISPR interference. J Bacteriol. 2021;203(12). Epub 2021/03/31. doi: 10.1128/JB.00565-20. PubMed PMID: 33782056.
14. Geisinger E, Mortman NJ, Dai Y, Cokol M, Syal S, Farinha A, et al. Antibiotic susceptibility signatures identify potential antimicrobial targets in the *Acinetobacter baumannii* cell envelope. Nature Communications. 2020;11(1):4522. doi: 10.1038/s41467-020-18301-2. PubMed Central PMCID: PMCPMC7481262.
