# Supplementary material for: Genome-wide phage susceptibility analysis in Acinetobacter baumannii reveals capsule modulation strategies that determine phage infectivity
Source: PLoS Pathog. 2023 Jun 8;19(6):e1010928. doi: 10.1371/journal.ppat.1010928 (PMC10249906; doi:10.1371/journal.ppat.1010928)
Supplement: S2 Table — (PDF) [file ppat.1010928.s012.pdf]

S2 Table. Spontaneous *gtr6* mutations conferring phage Loki resistance

gtr6 mutation	residue changes	strain(s), this study	KL	additional strains (Genbank)[b] strain name	accession	KL	additional strains (PATRIC)[b] strain name	accession	KL
440A$_{9\rightarrow8}$	V142Gfs*8	JBA149[a]	3	VB2107	CP051474.1	3	2015ZJAB25	WQMN01000013	3
		JBA151[a]	3	ABUH763	CP035051.1	22	2087	VMKD01000009	3
		JBA267[a]	3	AC29	CP007535.2	3	208510	VMJJ01000010	3
		JBA268[a]	3	AC30	CP007577.1	3	24860_4	JFDE01000009	22
				2018BJAB1	CP059351.1	3	4300STDY7045687	UFIZ01000012	3
				2018BJAB2	CP059350.1	3	4300STDY7045767	UFKE01000006	3
				2022CK-00784	CP117764.1	3	Ab83 strain D86	UEJK01000064	3
							C248	JAAZUG010000023	3
							PWa17_1044	JAJMQN010000011	3
							PWb12_715	JAJMPV010000012	3
							PWb48_2085	JAJMPN010000016	3
							PWb63_2087	JAJMPG010000011	3
							SP2107	JAAGSY010000002	3
384A$_{8\rightarrow7}$	M150Cfs*22	JBA261[a]	3	none			none		
425::IS*Aba13*	I131Ffs*6	EGA10	22	none			none		
		EGA65	22						

[a] ATCC 17978 background

[b] listed genomes had >75x sequencing coverage except C248 (24x), and no assembly anomalies
